# Myocardial segmental thickness variability on echocardiography is a highly sensitive and specific marker to distinguish ischemic and non-ischemic dilated cardiomyopathy in new onset heart failure

**DOI:** 10.1007/s10554-018-01515-3

**Published:** 2018-12-29

**Authors:** Chandra K. Katikireddy, Tushar Acharya

**Affiliations:** 1 Division of Cardiology, UCSF Fresno, 155 N Fresno St, Fresno, CA USA; 2 Division of Cardiology, VA Central California Health System, 2615 E. Clinton Ave, Fresno, CA 93703 USA

**Keywords:** Cardiomyopathy, Echocardiography, Myocardial segmental thickness variability

## Abstract

The aim of this study was to determine non-invasive diagnostic markers by echocardiography that differentiate ischemic dilated (ICM) from non-ischemic dilated cardiomyopathy (NICM) in patients with new onset heart failure. We identified 100 consecutive new heart failure patients with dilated cardiomyopathy (valvular etiology excluded). Clinical risk factors, medication history, serum biomarkers, ECG and echocardiographic variables were compared between the ICM and NICM groups (as confirmed by coronary angiography). Mean age, left ventricular size and ejection fraction were 56 years, 6.1 cm and 26% respectively. A total of 24% had ICM. Patients with ICM were older (65 vs. 53 years; P < 0.001). No significant difference was observed between ICM and NICM among 18 clinical variables, 7 laboratory tests, 6 EKG parameters and 10 of the 13 echocardiographic markers evaluated. Segmental wall thickness variability, regional wall motion abnormality and RV enlargement on echocardiogram (echo) differentiated ICM from NICM. Segmental thickness variability outperformed wall motion abnormality in diagnosing ICM with a sensitivity and specificity of 79.2 and 98.7% versus 62.5 and 84.2% respectively. RV enlargement was not sensitive but 90.6% specific for predicting NICM. Myocardial segmental thickness variability on echo, resulting from thinned infarcted or hibernating myocardium, is a highly sensitive and specific marker to differentiate ICM from NICM in new onset heart failure.

## Introduction

Echocardiography (echo) constitutes initial diagnostic work-up of patients presenting with new onset heart failure [1]. Further, echo helps to distinguish the pathological variants of cardiomyopathy [1, 2]. Patients with dilated cardiomyopathy and systolic dysfunction on echo often undergo non-invasive myocardial ischemic stress testing or invasive coronary angiogram to evaluate for coronary artery disease (CAD) as underlying etiology [3]. Non-invasive tests to detect CAD such as stress myocardial perfusion imaging or cardiac computed tomography may not be able to distinguish ischemic vs. non-ischemic cardiomyopathy with high degree of confidence. An echocardiographic marker to diagnose ICM and facilitate appropriate patient selection before an invasive procedure would be desirable. Older studies have used regional wall motion abnormality (RWMA) assessment as a diagnostic marker for ICM with conflicting results [4–6]. Coronary obstruction not only affects segmental wall motion but also wall thickness, with infarcted or hibernating myocardial segments frequently appearing thinner compared to surrounding healthy myocardium. The diagnostic utility of segmental wall thickness on echo has not been systematically evaluated. The objectives of this study were to determine non-invasive markers (clinical, EKG and echo parameters) that would reliably distinguish ICM from non-ischemic cardiomyopathy (NICM) in patients with new onset heart failure. We specifically evaluated segmental thickness variability (STV) as a novel non-invasive diagnostic marker in the evaluation of ICM and evaluated its diagnostic performance compared to the gold standard of angiography.

## Materials and methods

We retrospectively identified one hundred consecutive patients over a period of 3 years (2011–2014) admitted to a single center for new-onset systolic heart failure, who had dilated cardiomyopathy on echo (dilated left ventricle with an ejection fraction less than 40%), and underwent angiographic evaluation for ICM. After excluding those with (1) known history of dilated cardiomyopathy; (2) previous diagnosis of heart failure (hospital admission, emergency room, or clinic visit); (3) previous diagnosis of CAD (history of stable angina pectoris, acute coronary syndrome, positive stress testing, obstructive CAD on angiography, coronary intervention, or coronary artery bypass surgery); (4) acute coronary syndrome presentation; and (5) missing echo or angiographic data; 118 patients were identified. Further, those with (6) severe primary valvular heart disease that could contribute to LV dilatation (n = 2); (7) non-dilated cardiomyopathy (hypertrophic and restrictive cardiomyopathies with normal LV size) (n = 9); and (8) poor quality echo images precluding accurate segmental wall thickness/wall motion assessment (n = 7) were excluded. A total of 100 patients were included in the final analysis.

A thorough review of electronic medical records was conducted to collect demographics information, co-morbidities, clinical presentation, laboratory markers, pre-hospital medications, ECG, and the echo parameters at baseline. Myocardial segmental analysis was confirmed by the consensus read of two National Board of Echocardiography certified cardiologists. Investigators collecting baseline data and reviewing echo were blinded to the results of angiography. A single investigator, who was not involved in data collection, had access to angiography results. On the completion of data collection, this investigator categorized the patients into two groups (ICM and NICM) for statistical analysis. ICM was defined as severe (≥ 70%) stenosis in the left anterior descending or a multi-vessel coronary distribution. NICM had no angiographic stenosis, mild to moderate coronary stenosis (< 70% stenosis), or severe single-vessel stenosis in a coronary vessel other than the left main or left anterior descending artery [7] Degree of angiographic stenosis was based on the angiographer’s interpretation adjudicated by the study investigators.

**Fig. 1:**
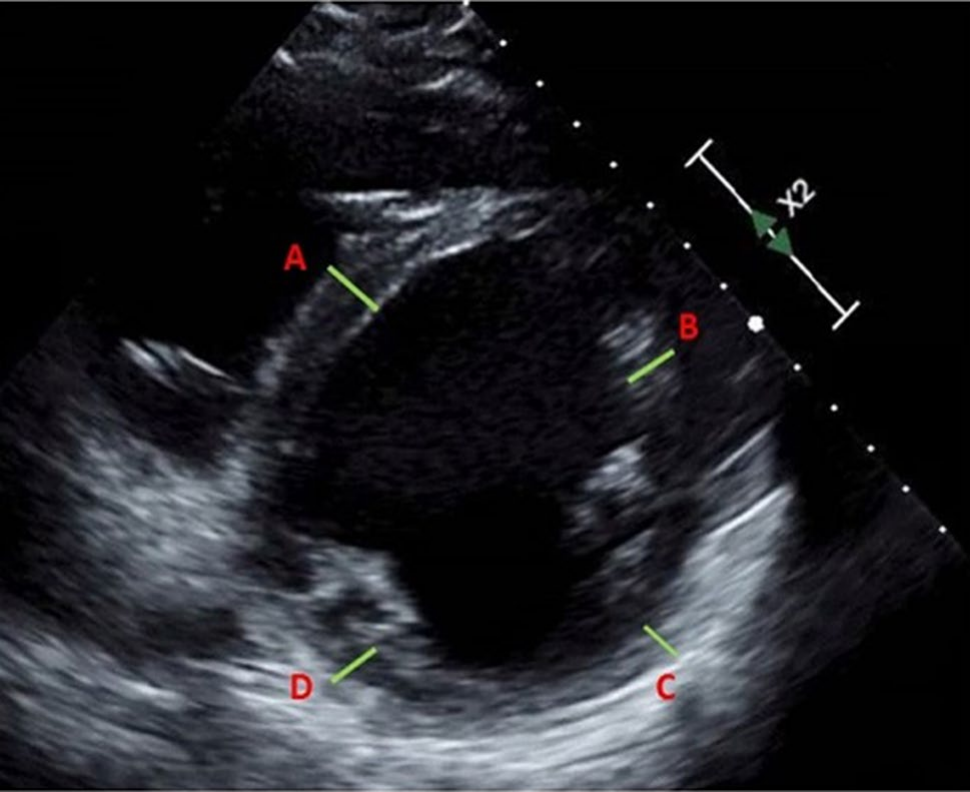
A case of non-ischemic dilated cardiomyopathy presenting as new onset heart failure. Short axis view of the left ventricle demonstrating uniform myocardial segmental thickness (9 ± 1 mm) and segmental thickness variability (STV) of > 0.65

**Fig. 2:**
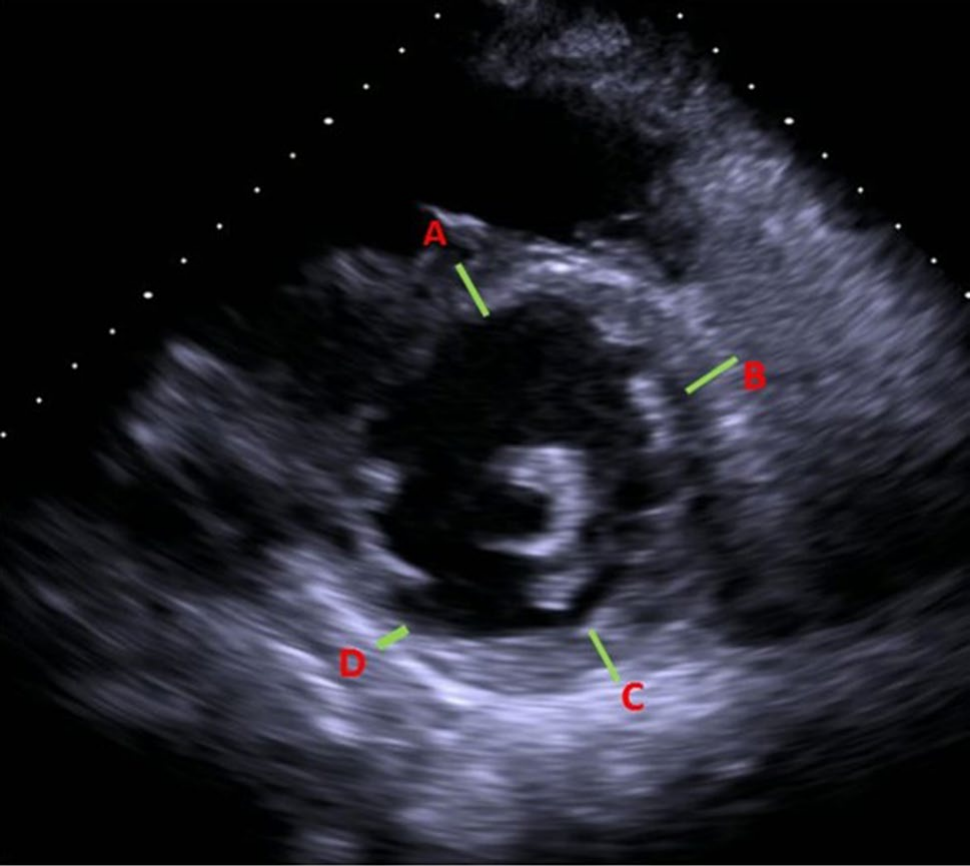
A case of new onset heart failure from ischemic dilated cardiomyopathy. Short axis view of the left ventricle illustrating thinned inferior wall (3 mm) and relatively thicker other segments (septum; 7 mm, anterior wall; 7 mm; lateral wall; 8 mm) in end diastolic phase with a segmental thickness variability (STV) of < 0.65

American heart Association’s sixteen-segment model was used for regional left ventricular (LV) wall analysis [8–10]. Basal, mid, and apical slices of the parasternal short-axis were used for segmental wall thickness analysis [8]. Short axis views were obtained orthogonal to the long axis of the LV to avoid skewed measurements from oblique cuts. Papillary muscles and the right ventricular (RV) septal band were systematically excluded wherever possible. Segmental thickness variability (STV) was measured in end-diastole, on a per-patient basis, and defined as a ratio of thinnest to the thickest wall segment in a different coronary artery territory [STV = thinnest wall segment (cm)/thickest wall segment (cm)]. Based on this definition, no variability (equally thick walls) would ideally yield a ratio 1. Based on preliminary analysis of the ICM cases, a cut-off value of 0.65 was used to define significant STV (Figs. 1, 2). Intra and inter-observer variability in the STV was estimated by two independent readers in 10 randomly selected subjects of ICM and 20 subjects of NICM.

RWMA was defined as per American Society of Echocardiography guidelines; hypokinesis, akinesis, dyskinesis, and aneurysmal segments were considered abnormal [8, 11]. Isolated septal WMA in patients with RV pressure/volume overload, left bundle branch block, or ventricular pacing were not reported as WMA from ICM.

Cardiac chamber quantification and function was estimated per ASE guideline statement [8]. Biplane measurements used to estimate LVEF. LV dilatation was defined as LV end diastolic diameter (2D echo − parasternal long axis view) > 5.2 cm in females and > 5.8 cm in males. In addition to visual estimation, RV size was measured by RV end diastolic basal diameter in RV—focused apical 4-chamber view. Tricuspid annular planar systolic excursion (TAPSE), tissue Doppler derived tricuspid lateral annular systolic velocity (S′) and 2D RV fractional area (FAC) change parameters were used to estimate RV systolic function.

### Statistical analysis

Demographic, clinical, laboratory, ECG, and echo parameters were compared between the ICM and NICM groups. Continuous variables were reported as mean and standard deviation and discrete variables as percentage. Analysis of variance (ANOVA) and Chi square tests were used to compare these variables, respectively. A two-sided P-value less than 0.05 was considered statistically significant. Variables that were significantly different between the comparison groups were evaluated for their sensitivity, specificity, negative and positive predictive values, and diagnostic accuracy for the diagnosis of ICM with invasive coronary angiography as the gold standard. Receiver operating characteristic curve analysis was performed. The intra-class correlation coefficient (ICC) was calculated as a measure of the intra- and inter-observer variability of the STV. A P value < 0.05 was considered significant.

SPSS 20 (IBM SPSS statistics for Windows version 20.0 Armonk, New York) was used for all statistical analysis.

## Results

The mean age of the study population was 56 years with 68% males. Mean LV size and LV ejection fraction were 6.1 cm and 26%, respectively. A total of 24% had ICM confirmed by coronary angiogram. Patients with ICM were older (65 vs. 53 years) (Table 1). As expected, hypertension, hyperlipidemia, diabetes, and stroke were more prevalent with ICM. None of the NICM patients had obstructive CAD. Alcoholism and illicit drug use were more common in the NICM group pointing towards other potential etiological mechanisms of heart failure. However, these differences in baseline comorbidities did not reach statistical significance and therefore were not used for diagnostic test evaluation. Clinical presentation of the two groups was not statistically different either. Patients with ICM had less favorable lipid profile with higher total cholesterol and low-density lipoprotein levels. They had statistically higher mean troponin but similar BNP levels on presentation. They were more likely to be on antiplatelet agents and statin medications. ECG parameters showed no statistical differences.

**Table 1:** Baseline characteristics of patients with new onset heart failure and dilated cardiomyopathy on echocardiography

	Non-ischemic dilated cardiomyopathy (n = 76)	Ischemic cardiomyopathy (n = 24)	P value
Age (years)	53.20 ± 14.43	65.17 ± 11.46	< 0.001
Male	51 (67.11)	17 (70.83)	0.086
Ethnicity			
Caucasian	11 (14.47)	5 (20.83)	0.456
Hispanic	38 (50.00)	7 (29.17)	
African-American	8 (10.53)	4 (16.67)	
Other	5 (6.58)	3 (12.5)	
Unknown	14 (18.42)	5 (20.83)	
Body mass index	29.26 ± 7.06	27.90 ± 5.90	0.392
Clinical presentation			
Shortness of breath/orthopnea/PND	59 (77.63)	23 (95.83)	0.064
Chest pain	26 (34.21)	12 (50.00)	0.228
Edema/weight gain	40 (52.63)	9 (37.50)	0.244
Duration of symptoms (days)	18.88 ± 26.43	17.46 ± 27.93	0.821
Atrial fibrillation at presentation	9 (11.84)	3 (12.50)	1.000
Heart rate at presentation	101.65 ± 19.92	98.83 ± 24.09	0.568
Systolic blood pressure at presentation	144.61 ± 27.84	136.75 ± 25.94	0.224
Diastolic blood pressure at presentation	93.72 ± 18.37	84.67 ± 20.43	0.043
Co-morbidities			
Hypertension	51 (67.11)	20 (83.33)	0.196
Hyperlipidemia	18 (23.68)	9 (37.50)	0.197
Diabetes mellitus	30 (39.47)	14 (58.33)	0.156
Chronic kidney disease	7 (9.21)	1 (4.17)	0.676
Chronic obstructive pulmonary disease	7 (9.21)	1 (4.17)	0.676
Stroke/transient ischemic attack	6 (7.89)	4 (16.67)	0.246
Peripheral vascular disease	2 (2.63)	1 (4.17)	0.565
Smoking	33 (43.42)	7 (29.17)	0.241
Alcoholism	17 (22.37)	4 (16.67)	0.775
Substance abuse	21 (27.63)	4 (16.67)	0.418
Laboratory markers			
Total cholesterol	137.20 ± 35.05	152.77 ± 43.23	0.095
Triglycerides	99.47 ± 53.72	105.82 ± 56.61	0.638
High density lipoprotein	36.36 ± 15.10	37.46 ± 12.33	0.760
Low density lipoprotein	81.09 ± 25.66	94.14 ± 33.68	0.062
BNP	1503.09 ± 1555.80	1284.33 ± 1207.51	0.556
Peak troponin	0.12 ± 0.21	0.83 ± 1.18	< 0.001
Hemoglobin A1C	6.99 ± 1.99	7.32 ± 2.14	0.526
Medications			
Aspirin	23 (30.26)	14 (58.33)	0.013
P2Y12 inhibitors	1 (1.32)	4 (16.67)	0.011
Beta-blockers	23 (30.26)	10 (41.67)	0.327
Angiotensin converting enzyme-inhibitors	19 (25.00)	13 (54.17)	0.012
Angiotensin receptor blockers	6 (7.89)	2 (8.33)	1.000
Thiazides	5 (6.58)	2 (8.33)	0.672
Calcium channel blockers	4 (5.26)	2 (8.33)	0.628
Other anti-hypertensive medications	10 (13.16)	3 (12.50)	1.000
Insulin	15 (19.74)	4 (16.67)	1.000
Oral hypoglycemic agents	9 (11.84)	5 (20.83)	0.314
Statins	15 (19.74)	12 (50.00)	0.007
EKG parameters			
Q waves	9 (12.00)	5 (20.83)	0.318
QRS duration	110.56 ± 22.80	116.17 ± 27.80	0.323
Poor R-wave progression	35 (46.67)	12 (50.00)	0.818
Left bundle branch block	5 (6.67)	1 (4.17)	1.000
Right bundle branch block	8 (10.67)	4 (16.67)	0.477
Non-specific IVCD	12 (16.00)	8 (33.33)	0.082
Echo parameters			
Left ventricular ejection fraction (%)	25.61 ± 6.90	26.04 ± 7.77	0.797
Diastolic dysfunction			
Grade 1	9/74 (12.16)	1 (4.35)	0.443
Grade 2–3	58/74 (78.38)	19 (82.61)	0.775
Left ventricular cavity size in diastole (cm)	6.10 ± 0.90	5.9817 ± 0.81	0.559
Left atrial diameter (cm)	4.62 ± 0.84	4.36 ± 0.47	0.146
Mean segmental thickness variability^a^	0.89 ± 0.08	0.63 ± 0.15	< 0.001
Segmental thickness variability	1 (1.32)	19 (79.17)	< 0.001
Regional wall motion abnormality	12 (15.79)	16 (66.67)	< 0.001
Right ventricular enlargement	29 (38.16)	3 (12.50)	0.023
Right ventricular dysfunction	36 (47.37)	9 (37.50)	0.483
RV systolic pressure	41.86 ± 13.81	39.44 ± 16.02	0.526
Ventricular thrombus	4 (5.26)	4 (16.67)	0.092
Mitral regurgitation			
Mild	46 (60.53)	16 (66.67)	0.638
Moderate to severe	17 (22.37)	6 (25.00)	0.786
Mechanism for moderate to severe mitral regurgitation			
Annular dilatation	5/15 (33.33)	5/6 (83.33)	0.063
Restrictive leaflet motion	10/15 (66.67)	1/6 (16.67)	
Pericardial effusion	18 (23.68)	8/24 (33.33)	0.424

*PND* paroxysmal nocturnal dyspnea, *IVCD* intra-ventricular conduction delay

^a^Segmental thickness variability (%) = thinnest wall segment (cm)/thickest wall segment (cm)

Mean LV dimensions and systolic function was comparable between the two groups. Similarly, diastolic function and LA dimensions did not provide significant distinction. RV enlargement was more frequently seen in NICM patients (38 vs. 13%; P = 0.023) but RV systolic function and RV systolic pressures were similar. Using the pre-specified cut-off, significant STV was observed in 79% of ICM (mean STV 63%) and only 1% of NICM (mean STV 89%) (P < 0.001). ICM patients also had more RWMA than NICM patients (67 vs. 16%; P < 0.001).

**Fig. 3:**
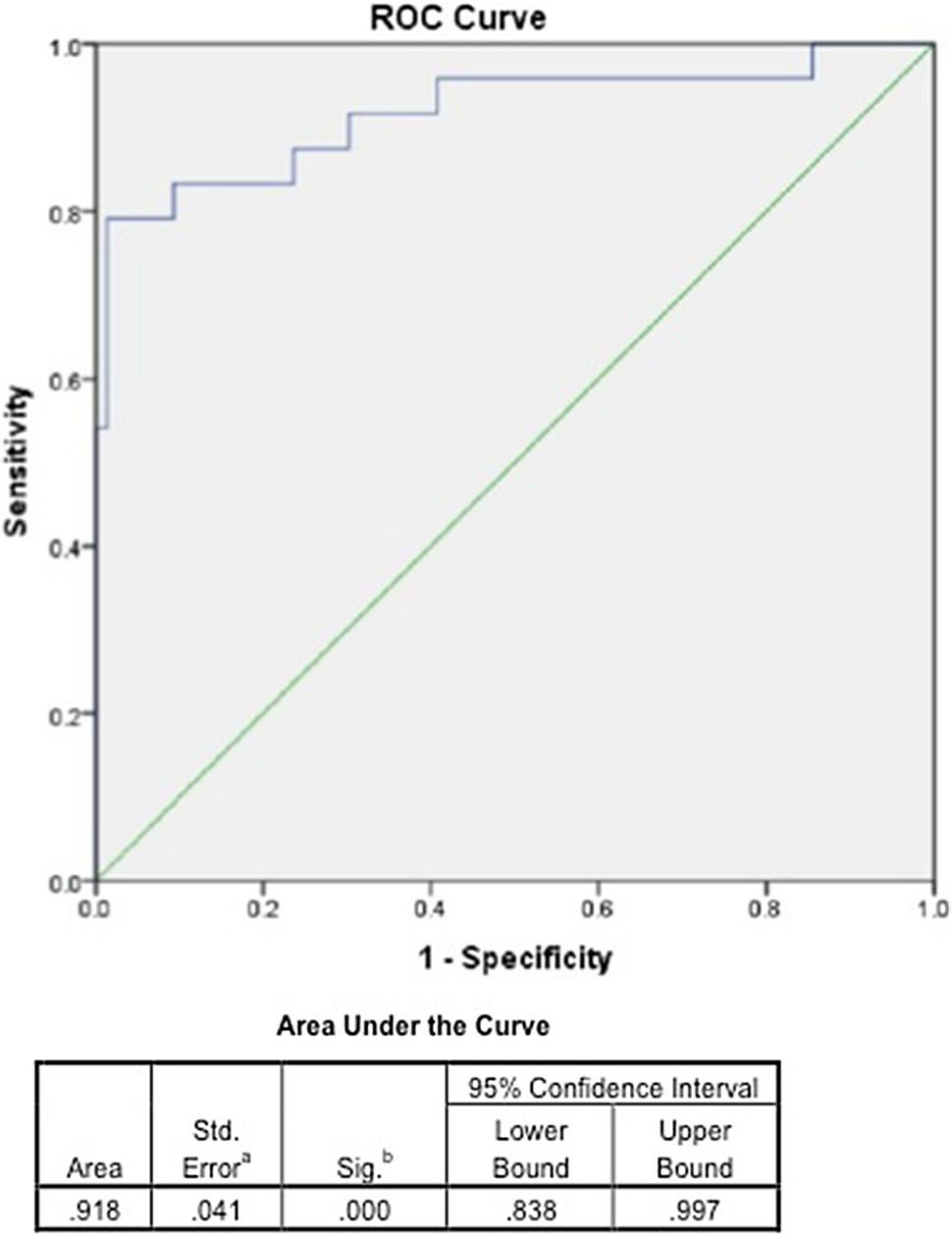
Receiver operator curve analysis showing the performance of segmental thickness variability ratio from echocardiography to distinguish ischemic from non-ischemic dilated cardiomyopathy as confirmed by coronary angiogram

Diagnostic test evaluations for STV, RWMA, and RV enlargement are elaborated in Table 2. STV outperformed RWMA and RV size in diagnosing ICM with a sensitivity and specificity of 79.2% and 98.7%. RWMA was more specific (84.2%) than sensitive (66.7%). The most sensitive (91.7%) way of identifying ICM on echo was to use the presence of either STV or RWMA (negative predictive value 96.9%) as a diagnostic marker. On the other hand, the presence of both STV and RWMA made the test 100% specific for ICM with a positive predictive value of 100%. STV had the overall highest diagnostic accuracy. Results of the receiver operating characteristic curve are shown in Fig. 3. Intra and inter observer variability for STV measurement in ICM and NICM were good. The ICC values were 0.90 (95% CI 0.86–0.98), 0.92 (95% CI 0.90%–0.98) for intra observer variability and 0.84 (95% CI 0.78–0.88), 0.89 (95% CI 0.82–0.92) for inter-observer variability in ICM and NICM respectively.

**Table 2:** Diagnostic test evaluation for select echocardiographic predictors of ischemic cardiomyopathy

	Sensitivity (%)	Specificity (%)	Positive predictive value (%)	Negative predictive value (%)	Accuracy (%)
Segmental thickness variability (STV)	79.17	98.68	95	93.75	94
Regional wall motion abnormality (RWMA)	66.67	84.21	57.14	88.89	80
STV or RWMA	91.67	82.89	62.86	96.92	85
STV and RWMA	54.17	100	100	87.36	89
Normal right ventricular size	87.5	38.16	30.88	90.63	50

## Discussion

Heart failure is a frequently diagnosed condition and a common cause of significant morbidity and mortality [12]. A thorough initial work up to identify the underlying etiology can have prognostic and therapeutic implications. Certain phenotypes, like ICM, have higher mortality than other groups [1, 13, 14].

To distinguish ICM from NICM, ACCF/AHA heart failure guidelines suggest that coronary angiogram be performed in patients presenting with de novo heart failure (Class IIA, level of evidence: C) [15]. However, the use of angiography as a ‘rule-out’ tool has low diagnostic yield in these patients. Though ICM is the most prevalent form of dilated cardiomyopathy, the majority of these patients present with angina or myocardial infarction and only a few as de novo heart failure [16, 17]. In a study by Felker et al. among 1230 heart failure patients referred for endomyocardial biopsy, less than 10% were found to have ICM [1, 14]. In a recent study comparing MRI and angiography to distinguish ICM and NICM, ICM was reported in 22–26% of de novo HF patients [18]. In the current study, we found ICM, as defined by angiogram, in 24% of new heart failure patients, falling within the general range of previously published data. Given this low incidence, finding a reliable pre-catheterization test to stratify patients based on likelihood of having ICM could prevent invasive testing in many. Low probability patients could instead be evaluated with a non-invasive modality (also a Class IIA recommendation) to exclude ischemic etiology.

In this study, we evaluated 18 clinical variables, 7 laboratory tests, 6 EKG parameters, and 13 echocardiographic markers with the goal of finding a diagnostic market that would reliably distinguish ICM from NICM (Table 1). As expected, patients with ICM were significantly older mirroring the prevalence of CAD. They had more prevalent clinical risk factors like hypertension, diabetes and hyperlipidemia (with higher levels of total cholesterol and LDL). However, these differences did not reach statistical significance and thus were insufficient in providing classification advantage in the 100 patients studied. Similarly, EKG findings could not distinguish the two groups. These findings suggest that a pre-test probability evaluation for underlying coronary artery disease based on risk factor and clinical presentation may not be feasible in new heart failure patients.

Wall motion is a dynamic motion of the ventricular wall in systole and diastole that may be impaired by ischemia/infarction resulting in RWMAs. Wall thickness, on the other hand is more objective as measured at a single point in time, such as end systole or end-diastole. We measured end-diastolic wall thickness in this study as end-diastole represents true resting physiologic state of the myocardium. End-systolic measurement may be more prone for artefactual errors due to inclusion of the adjacent structures such as chordae and papillary muscle. Mechanisms of ischemic wall thinning are well described. Myocardial infarction causes myocyte necrosis and replacement with collagen scar resulting in thinning of the wall in the distribution of culprit coronary vessel [19, 20]. Similarly, in severe ischemia leading to hibernating myocardium, the wall in the affected territory thins out from loss of contractile elements and dedifferentiation, whereas surrounding segments may undergo compensatory hypertrophy and remodeling [21–24]. The resulting difference in segmental thickness is more obvious and may be unique to ICM and can be evaluated and interpreted as the STV ratio. We found an STV ratio of < 0.65 to have excellent predictive value for ICM with 92% area under the curve (Fig. 1).

We systemically evaluated pre-angiographic non-invasive markers in new onset heart failure with dilated cardiomyopathy and found the presence of STV, RWMA, and normal RV size on echo to correspond to coronary vascular etiology. Among these parameters, STV by itself was the most sensitive and specific diagnostic marker of ICM with a predictive accuracy of 94%. Using STV in conjunction with RWMA could further increase the diagnostic test performance of STV. Visualization of either STV or RWMA could increase the sensitivity of identifying ICM, whereas the presence of both STV and RWMA in a patient made the test 100% specific for ICM. RV enlargement was found to be a sensitive but non-specific marker for NICM (Table 2).

To our knowledge, this is the first study to report STV on echocardiography as a diagnostic marker of ICM. RWMA on the other hand has been studied previously, albeit with disparate findings [4–6]. In the current study, we found regional wall motion abnormalities to be 67% sensitive and 84% specific in diagnosing ICM. These differences highlight the subjective nature of wall motion analysis with significant inter-user variability. Additionally, it is widely known that RWMA can result not only from coronary ischemia but a number of other conditions such as primary myopericardial disease [25–29]. To increase specificity for ICM, we did not report septal abnormalities from right ventricular pressure/volume overload, left bundle branch block or ventricular pacing. Even still, RWMA was outperformed by STV.

Our study has a few limitations. The use of coronary angiography as the reference standard for distinguishing the cardiomyopathies may be a potential limitation. Further studies are needed to assess the reproducibility of this single center experience with a small number of patients and overcome potential reader bias in the interpretation of segmental wall analysis. Our study findings are not applicable to patients with suboptimal echo image quality from poor ultrasongraphic windows and technique. This may be overcome partially with the use of echocardiographic contrast agent. Prospective validation of this retrospective study would be desirable.

## Conclusions

Dilated cardiomyopathy is a common disease process in patients presenting with new onset heart failure. In this patient population, STV as estimated by transthoracic echocardiography can accurately distinguish ICM from NICM. RWMA in isolation is a less accurate marker to make this distinction. Used in conjunction, these two parameters can reliably delineate ICM from NICM.

These echocardiographic markers to distinguish the underlying etiology of new onset heart failure may provide additional guidance to cardiac imagers and clinicians and guide appropriate therapy.

## Abbreviations

Echo: Echocardiogram
CAD: Coronary artery disease
ICM: Ischemic cardiomyopathy
LV: Left ventricle
NICM: Non-ischemic cardiomyopathy
RWMA: Regional wall motion abnormality
STV: Segmental wall thickness variability
RV: Right ventricle
